# Small Molecules from Medicinal Plant *Iris tectorum* as Histidine Kinase Inhibitor to Resensitize β-Lactam-Resistant *Escherichia coli*

**DOI:** 10.3390/molecules30030663

**Published:** 2025-02-03

**Authors:** Youqi Ji, Yinhuan Wang, Zhangkai Xu, Danlei Chen, Zhendi Yu, Qingyi Shao, Xin Hong, Zishu Liu, Dongqing Cheng

**Affiliations:** 1 School of Medical Technology and Information Engineering, Zhejiang Chinese Medical University, Hangzhou 310053, China; jiyouqi_gym@163.com (Y.J.); xchxzk@163.com (Z.X.); danleichen1@outlook.com (D.C.); 202111116011021@zcmu.edu.cn (Z.Y.); shaoqingyieve@163.com (Q.S.); 2 Zhejiang Institute for Food and Drug Control, Hangzhou 310052, China; wangyinhuan@zjyj.org.cn; 3 Department of Green Pharmaceutical Collaborative Innovation Center, School of Pharmacy, Zhejiang University of Technology, Hangzhou 310014, China; 221122070290@zjut.edu.cn; 4 College of Environmental and Resource Sciences, Zhejiang University, Hangzhou 310058, China

**Keywords:** *Iris tectorum*, *Escherichia coli*, β-lactam resistance, histidine kinase, phosphorylation, molecular docking

## Abstract

Background: Due to the widespread use of broad-spectrum antibiotics, the problem of antibiotic resistance has become an increasingly serious global threat. One of the key mechanisms of *Escherichia coli* resistance to beta-lactam antibiotics is the production of beta-lactamase enzymes, which poses a dilemma for clinicians in selecting antibiotics when faced with resistant bacterial infections. However, research on the reversal of bacterial resistance is limited. Methods: This study involved the preparation of *Iris tectorum* extract and detection of its effects on antibiotics sensitivity, extended-spectrum beta-lactamase (ESBL) gene expression, and histidine kinase phosphorylation levels in β-lactam antibiotic-resistant *Escherichia coli*. Additionally, analyses of the active ingredients of *Iris tectorum* extract were conducted with a liquid chromatography–mass spectrometer, and the binding sites were predicted by molecular docking. Results: *Iris tectorum* extract could restore the sensitivity of *Escherichia coli* to beta-lactam antibiotics and reduce the expression levels of ESBL genes and histidine phosphorylation levels. The active ingredients of *Iris tectorum* extract may be irigenin and tectorigenin, and these two small molecules could bind to histidine kinase to inhibit phosphorylation. Conclusions: *Iris tectorum* extract may serve as an antibiotic adjuvant, restoring the sensitivity of antibiotic-resistant bacteria by inhibiting histidine kinase phosphorylation, thereby alleviating the problem of *Escherichia coli* resistance to β-lactam antibiotics.

## 1. Introduction

The advent and application of antibiotics laid the foundation for the development of modern medicine; however, the abuse of antibiotics has led to the emergence of antibiotic resistance, posing a paramount public health concern worldwide [1,2,3]. The origins of antibiotic resistance remained a subject of debate. Some researchers believe that antibiotic resistance has existed since ancient times, and bacteria inherently possess certain antibiotic resistance genes (ARGs) [4,5]. However, other researchers believe that antibiotic resistance is acquired, with the excessive use of antibiotics in clinical settings, as well as in agriculture and animal husbandry, exerting selective pressure that facilitates the emergence of antibiotic resistance [3,6,7]. Of greater concern, antibiotic resistance is growing faster than the development of new antibiotics [8]. According to the forecast of the World Health Organization, the annual global mortality attributed to antibiotic-resistant bacterial infections is anticipated to surge to 10 million by 2050, which is very frightening [9]. In the face of this dilemma, despite continuous calls from governments and public health organizations for the development of novel antibiotics, and the introduction of encouraging policies and investments, the process of bringing new antibiotics to clinical use remains arduous. This low return on investment often leads pharmaceutical companies to abandon their antibiotic research programs [10].

Therefore, the emergence of antibiotic adjuvants has provided new ideas for combatting antibiotic resistance. Antibiotic adjuvants refer to compounds or formulations that enhance antibiotic efficacy or target antibiotic-resistance mechanisms. Antibiotic adjuvants can not only effectively enhance the efficacy of existing antibiotics but also enable the reuse of outdated antibacterial compounds, which can reduce the demand for new antibiotics and thus lower the cost of new drug development [11]. Moreover, since these compounds or formulations are not antibiotics inherently, the selective pressure is much lower compared to traditional antibiotics [12]. For example, the anti-diarrhea drug loperamide has been identified as an adjuvant of minocycline. Loperamide can promote the absorption of minocycline by drug-resistant bacteria and improve the aggregation of antibiotics in cells to kill bacteria [13].

At present, many studies have shown that some traditional Chinese medicines have significant antibacterial and bactericidal effects and also show antibacterial activity through a synergistic effect when used in combination with antibiotics [14]. *Iris tectorum* (*I. tectorum*) is a traditional Chinese medicine that has had great medicinal value in the treatment of bronchitis, internal injuries, rheumatism, and some infectious diseases since ancient times [15]. Our study found that *I. tectorum* extract can act on drug-resistant *Escherichia coli* (*E. coli*) and reduce its resistance to β-lactam antibiotics. According to the monitoring report of the China Antimicrobial Resistance Surveillance System, 4,928,509 strains of drug-resistant bacteria were included in the analysis in 2022, of which *E. coli* accounted for the highest proportion (1,026,214 strains; 20.8%). The main mechanism of antibiotic resistance in *E. coli* is extended-spectrum beta-lactamase (ESBL) production [16]. Various types of ESBLs have been classified based on the homology of their plasmid-encoded genes including CTX-M, TEM, SHV, and KPC. CTX-M was more effective against cefotaxime and ceftriaxone than ceftazidime. Mutations in TEM- and SHV-derived ESBL genes lead to alterations in enzyme activity, resulting in the decreased efficacy of broad-spectrum cephalosporins. Additionally, the KPC enzyme is capable of hydrolyzing carbapenem antibiotics, posing significant challenges in the treatment of drug-resistant bacteria [17,18,19]. The two-component signaling system (TCSS) plays a significant role in the production of ESBLs in *E. coli*. In order to sense and respond to antibiotic pressure, the TCSS has a histidine kinase (HK) unit to sense the environment and a related response regulator protein (RRP) unit to receive the signal and translate it into gene expression changes [20,21,22]. The regulation of the TCSS on bacterial resistance depends on the phosphorylation of histidine kinase. Therefore, new antibiotics or antibiotic adjuvants can be developed to target bacterial protein kinases [23].

In this study, we investigated the changes in antibiotic sensitivity of beta-lactam antibiotic-resistant *E. coli* after pretreatment with *I. tectorum* extract and explored the relationship between ESBL gene expression and bacterial protein phosphorylation. The results of this study will expand the application of traditional Chinese medicine resources and contribute to the development of antibiotic adjuvants.

## 2. Results

### 2.1. I. tectorum Extract Could Reduce the MIC of Antibiotics Against E. coli

The MIC of ampicillin after simultaneous treatment with 96.3 μg/mL *I. tectorum* extract and ampicillin was lower than 15.6 μg/mL; the MIC of ampicillin decreased from 2000.0 μg/mL after simultaneous treatment with 48.1 μg/mL *I. tectorum* extract and ampicillin to 125.0 μg/mL, and the MIC of ampicillin was still 2000.0 μg/mL after simultaneous treatment with 19.3 μg/mL *I. tectorum* extract and 3.9 μg/mL ampicillin (Table 1). The fractional inhibitory concentration index (FICI) of *I. tectorum* extract was 0.3. The obtained values were used to categorize the interactions between the two drugs tested in combination. The FICI values were defined as synergy for FICI ≤ 0.5, additivity for 0.5 < FICI ≤ 1, indifference for 1 < FICI ≤ 2, and antagonism for FICI > 2 [24]. Therefore, when the *I. tectorum* extract and ampicillin acted on drug-resistant bacteria at the same time, there was a synergistic effect, which could enhance the sensitivity of drug-resistant bacteria to ampicillin.

**Figure 1:**
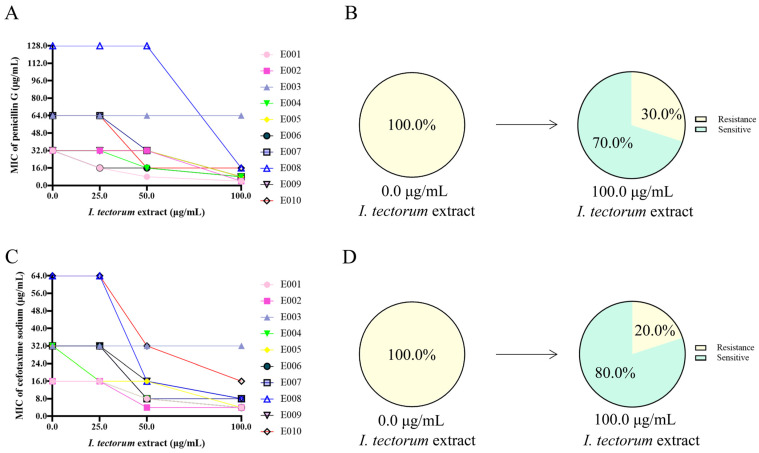
Resensitization of β-lactam-resistant *E. coli* under the treatment of *I. tectorum* extract. (**A**) Under the treatment of *I. tectorum* extract, the MIC of penicillin G against drug-resistant bacteria decreased. (**B**) At the final concentration of 100.0 μg/mL, penicillin G resistance was reversed in 70% of *E. coli*. (**C**) Under the treatment of *I. tectorum* extract, the MIC of cefotaxime sodium against drug-resistant bacteria decreased. (**D**) At the final concentration of 100.0 μg/mL, cefotaxime sodium resistance was reversed in 80% of *E. coli*.

After co-incubation with different concentrations of *I. tectorum* extract for 0.5 h, the resistance of the strains to penicillin G and cefotaxime decreased to varying degrees (Figure 1A,C). Under the action of 100.0 μg/mL of *I. tectorum* extract, 70% of the strains regained their sensitivity to penicillin G, and 80% of the strains regained their sensitivity to cefotaxime (Figure 1B,D).

### 2.2. I. tectorum Extract Downregulated the Transcript Levels of ESBLs Genes

**Figure 2:**
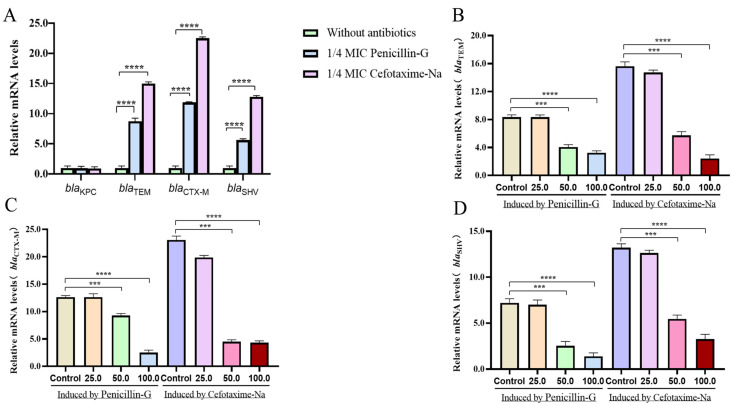
*I. tectorum* extract inhibited the expression levels of ESBLs gene. (**A**) After induction with 1/4MIC antibiotics, the expression of ESBL genes in drug-resistant bacteria were significantly upregulated. (**B**) After being induced by antibiotics, the expression of *bla*_TEM_-resistant bacteria could be inhibited by 50.0 μg/mL and 100.0 μg/mL of *I. tectorum* extract. (**C**) After being induced by antibiotics, the expression of *bla*_CTX-M_-resistant bacteria could be inhibited by 50.0 μg/mL and 100.0 μg/mL of *I. tectorum* extract. (**D**) After being induced by antibiotics, the expression of *bla*_SHV_-resistant bacteria could be inhibited by 50.0 μg/mL and 100.0 μg/mL of *I. tectorum* extract. The horizontal axis of (**B**–**D**) represented the concentration of *I. tectorum* extract, while Control represented the blank control group. ESBLs-mRNAs levels were statistically analyzed using a *t* test; “***” means *p* < 0.001, and “****” means *p* < 0.0001.

Four strains, namely E001 (*bla*_TEM_), E002 (*bla*_CTX-M_), E003 (*bla*_KPC_), and E004 (*bla*_SHV_), which were resistant to penicillin G and cefotaxime sodium and contained only a single ESBL gene were selected. There was a significant upregulation in the transcript levels of *bla*_TEM_, *bla*_CTX-M_, and *bla*_SHV_ induced by 1/4 MIC of penicillin G and cefotaxime sodium (Figure 2A) (*p* < 0.0001).

After co-incubation with different concentrations of *I. tectorum* extract for 0.5 h, the transcription level of *bla*_TEM_ induced by 1/4 MIC of penicillin G and cefotaxime decreased by 2–6 fold (Figure 2B), the transcription level of *bla*_CTX-M_ decreased by 1–5 fold (Figure 2C), and the transcription level of *bla*_SHV_ decreased by 2–5 folds (Figure 2D).

### 2.3. I. tectorum Extract Reduced Histidine Kinase Phosphorylation Levels in the TCSS

**Figure 3:**
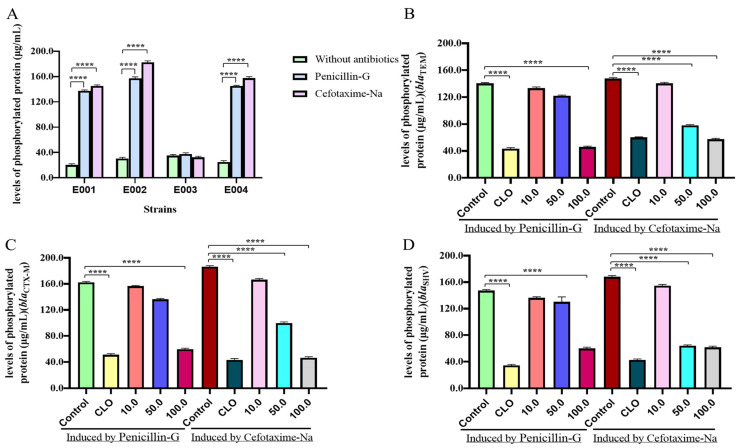
*I. tectorum* extract reduced histidine kinase phosphorylation levels in the TCSS. (**A**) After antibiotic induction, protein phosphorylation levels of E001 (*bla*_TEM_), E002 (*bla*_CTX-M_), and E004 (*bla*_SHV_) were significantly increased, while E003 (*bla*_KPC_) had no significant difference. (**B**) After induction of penicillin G, the protein phosphorylation of *bla*_TEM_-resistant bacteria could be inhibited by 100.0 μg/mL of *I. tectorum* extract, while that of cefotaxime sodium could be inhibited by 50.0 μg/mL and 100.0 μg/mL of *I. tectorum* extract. (**C**) After induction of penicillin G, the protein phosphorylation of *bla*_CTX-M_-resistant bacteria could be inhibited by 100.0 μg/mL of *I. tectorum* extract, while that of cefotaxime sodium could be inhibited by 50.0 μg/mL and 100.0 μg/mL of *I. tectorum* extract. (**D**) After induction of penicillin G, the protein phosphorylation of *bla*_SHV_-resistant bacteria could be inhibited by 100.0 μg/mL of *I. tectorum* extract, while that of cefotaxime sodium could be inhibited by 50.0 μg/mL and 100.0 μg/mL of *I. tectorum* extract. The horizontal axis of (**B**–**D**) represented the concentration of *I. tectorum* extract, with Control as the blank control group and CLO as the positive control group. Protein phosphorylation levels were statistically analyzed using a *t* test; **** means *p* < 0.0001.

The phosphorylation levels of E001 (*bla*_TEM_), E002 (*bla*_CTX-M_), E003 (*bla*_KPC_), and E004 (*bla*_SHV_), which were induced by penicillin G and cefotaxime sodium, increased significantly, with the exception of E003 (Figure 3A) (*p* < 0.0001).

After co-incubation with different concentrations of *I. tectorum* extract for 0.5 h, the histidine kinase phosphorylation levels of E001 (*bla*_TEM_) induced by 1/4 MIC penicillin G and cefotaxime decreased by 1–3-fold (Figure 3B), E002 (*bla*_CTX-M_) decreased by 1–4-fold (Figure 3C), and E004 (*bla*_SHV_) decreased by more than 2-fold (Figure 3D).

The HK inhibitor Closantel (132.5 μg/mL) was used as the positive control, and there was a significant downregulation in histidine kinase phosphorylation levels of *bla*_TEM_, *bla*_CTX-M_, and *bla*_SHV_ induced by 1/4 MIC of penicillin G and cefotaxime sodium (Figure 3) (*p* < 0.0001).

### 2.4. Analysis of I. tectorum Extract Components

**Figure 4:**
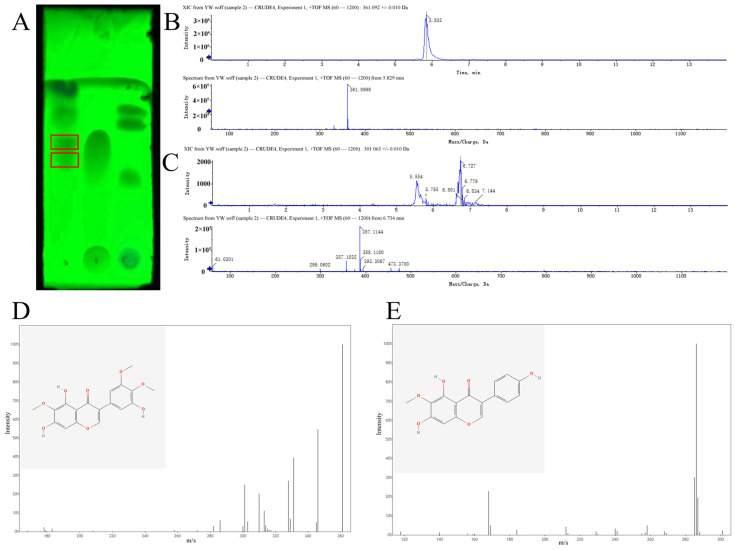
Determination of components in *I. tectorum* extract. (**A**) TLC test results. From left to right are the *I. tectorum* extract, flavonoid standard, and crude drug extract. The red box in the figure indicates the flavonoids in the *I. tectorum* extract. (**B**) One component was detected by LC-MS at 5.829 min and was determined as irigenin. The arrows indicated that peaks below this value didn’t display numerical values. (**C**) One component was detected by LC-MS at 6.734 min and was determined as tectorigenin. The arrows indicated that peaks below this value didn’t display numerical values. (**D**) The peak spectrum and structural formula of irigenin after analysis. The structural formula can be downloaded from PubChem. (**E**) The peak spectrum and structural formula of tectorigenin after analysis. The structural formula can be downloaded from PubChem.

Using the flavonoid extraction method, we obtained 154.0 mg of extract from 200 g of dried rhizomes. With 80% ethanol as a blank, the absorbance was measured at 266.5 nm wavelength, and the standard curve was drawn with the absorbance value of the sample as the vertical coordinate (A) and the concentration as the horizontal coordinate (C), as shown in Supplementary Materials Figure S1. Moreover, the 154.0 mg crude extract contained 36% total flavonoids. Thin-layer chromatography (TLC) showed that there were two types of flavonoids in *I. tectorum* extract (Figure 4A). These two substances were identified as irigenin and tectorigenin in the liquid chromatography–mass spectrometry (LC-MS) assay (Figure 4B–E).

### 2.5. Molecular Docking Sites

**Figure 5:**
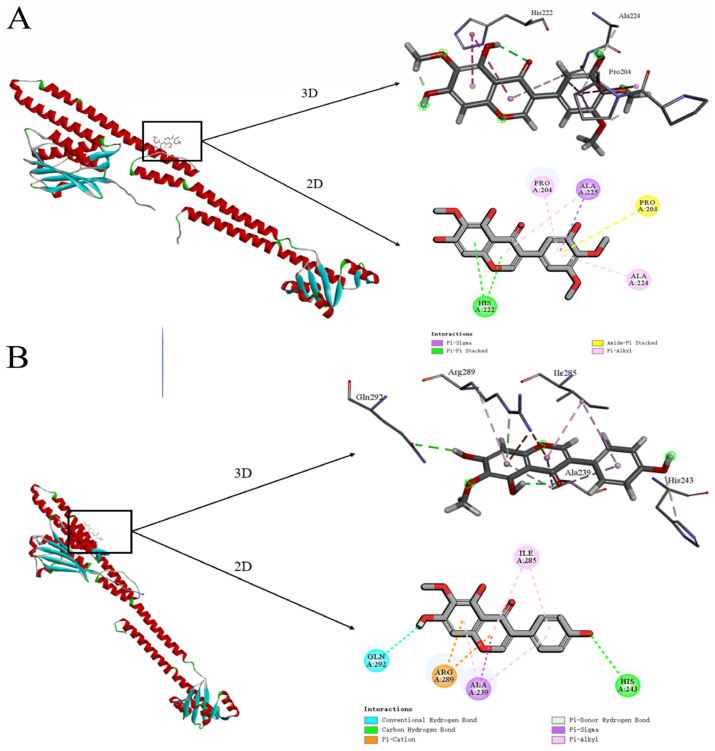
(**A**) Irigenin molecular docking schematic diagram. (**B**) Tectorigenin molecular docking schematic diagram.

Through molecular docking calculations, the binding sites of irigenin and tectorigenin with histidine kinase protein were identified. Irigenin bound to proline 203 of histidine kinase through Amid-Pi Stacked, to proline 204 vialled Pi-Alkyl, to histidine 222 through Pi-Pi Stacked, to alanine 224 vialled Pi-Alkyl, and to alanine 225 through Pi-Alkyl and Pi-Sigma (Figure 5A). On the other hand, tectorigenin bound to alanine 239 through both Pi-Alkyl and Pi-Sigma, to histidine 243 vialled a Carbon Hydrogen Bond, to isoleucine 285 through Pi-Alkyl, to arginine 289 through a Pi-Cation, and to glutamine 292 through a Conventional Hydrogen Bond (Figure 5B).

## 3. Discussion

In clinical treatment, doctors usually prescribe two or more antibiotics, which can expand the treatment scope, cover all potential bacterial infections, and avoid ineffective treatment caused by bacterial resistance, improving the prognosis of patients [25]. Complementary to combination therapy with multiple antibiotics, the use of bioactive chemical entities, such as antibiotic adjuvants, to treat resistant bacterial infections is also considered as an effective approach [11]. Antibiotic adjuvants can be divided into four categories based on their mode of action: resistance inhibitors, membrane saboteurs, signaling inhibitors, and immune enhancers. Among them, the TCSS is one of the most representative signaling systems targeted by signal inhibitors [26].

**Figure 6:**
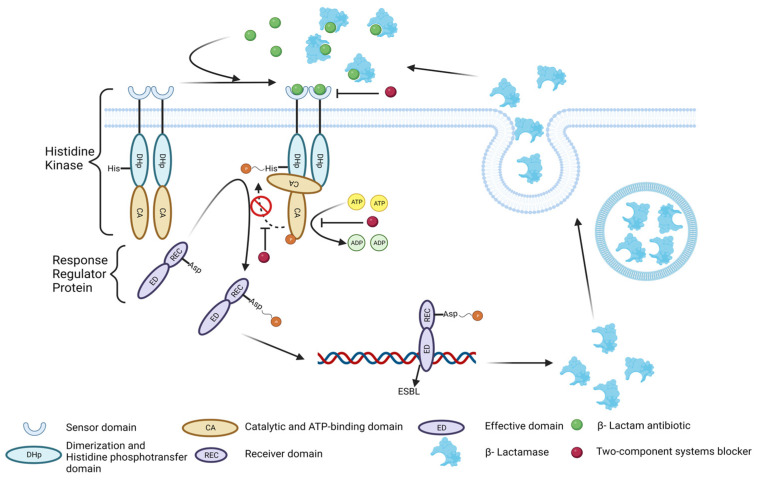
Mechanism diagram of the TCSS. The arrows in the figure represented the operation process of TCSS, including receiving stimuli, activation, site binding, gene transcription, protein translation, protein transport, and efflux. This figure was drawn through BioRender.

The TCSS is an important signal transduction system that is associated with bacterial resistance. According to the amino acid types of their phosphorylated substrate proteins, their protein kinases can be divided into three categories: serine threonine kinase (SK), tyrosine kinase (TK), and HK, among which HK is the main regulator of *E. coli* gene expression. When β-lactam antibiotics reach the surface of bacteria as a stimulus signal, they can bind to HK receptors and transmit the signal into the cell. Thus, histidine in the catalytic site of intracellular histidine kinase can be activated by ATP phosphorylation. Subsequently, RRP can transfer the phosphate group from HK histidine residues to its own aspartic acid residues, making it phosphorylated and activated, thus obtaining the ability to bind to the ESBLs gene promoter and regulate its expression, reversing bacterial resistance to β-lactam antibiotics (Figure 6) [27,28,29,30]. Among these steps, self-phosphorylation activation of HK is a key step in the initiation of the TCSS [28]. In addition, we found that 1/4 MIC of penicillin G and cefotaxime sodium significantly enhanced the protein phosphorylation level of *E. coli*. This result indicates that the resistance of *E. coli* to β-lactam antibiotics is regulated by the TCSS.

At present, it has been reported that most of the preparations produced can block the TCSS by inhibiting HK [31]. Keith et al. (2000) reported that closantel, ofloxacin, and some compounds that were synthesized by themselves could inhibit the HK autophosphorylation of the TCSS in *E. coli* [32]. Yamamoto et al. (2001) have verified that HK inhibitors, such as synthetic imidazole, zerumbone derivatives, and aranorosinol B are effective antibacterial agents against *Bacillus subtilis* [33]. However, none of the identified inhibitors have been utilized in clinical practice, and none any of these compounds are undergoing clinical trials. This is because of the poor bioavailability of many inhibitors, stemming from their highly hydrophobic properties, as well as the potential for some inhibitors to induce hemolysis. Furthermore, it remains uncertain whether the ability to inhibit HK, as observed in assays, is responsible for their antimicrobial activities or if the killing of bacteria is unrelated to HK inhibitory activity [28,33].

In the treatment of multidrug-resistant bacteria, apart from the combined use of different antibiotics, the use of antibiotics combined with traditional Chinese medicines has been demonstrated in various studies [34]. *I. tectorum* contains abundant isoflavones, mainly including tectoridin, tectorigenin, wild iridoin, iridoin A/B, etc. [35]. It has significant biological activities, such as antioxidant, anti-aging, anti-tumor, anti-malaria, and anti-tuberculosis activities and reducing lipid peroxidation and preventing cardiovascular diseases and osteoporosis [36]. Previous studies have shown that flavonoids have various physiological activities and pharmacological effects such as anti-free radical, anti-tumor, antioxidant, antibacterial, antiviral, anti-inflammatory, etc. Iris drugs have stronger overall antibacterial and free radical scavenging abilities compared to other traditional Chinese medicines containing flavonoids as reported in the literature [37]. We speculate that *I. tectorum* extract may act as a HK inhibitor, and it can combine with antibiotics to work against bacterial resistance. We selected clinically isolated *E. coli* that were resistant to β-lactam antibiotics to explore the relationship between the TCSS and β-lactamase genes, study the effect of antibiotics on the expression of a single β-lactamase gene, and understand whether *I. tectorum* extract can inhibit HK phosphorylation and change antibiotic susceptibility against multidrug-resistant *E. coli*. We selected E001 (*bla*_TEM_), E002 (*bla*_CTX-M_), E003 (*bla*_KPC_), and E004 (*bla*_SHV_), which contain only one β-lactamase gene, as the research objects. We selected closantel, an HK inhibitor, as the positive control. To explore the effect of antibiotics and *I. tectorum* extract on protein phosphorylation levels in bacteria, we used a protein phosphorylation enrichment kit, a protein phosphorylation level detection kit, and an HK-specific inhibitor inhibition test. Kinases are biochemical molecules that transfer phosphate groups from high-energy donor molecules to specific target molecules; this process is called phosphorylation. We used histidine kinase inhibitors as controls to confirm that HK phosphorylation of histidine kinase was inhibited in the experiment. We analyzed HK activation and its response to regulatory proteins by detecting a variety of protein phosphorylation levels in bacteria. Penicillin G was selected for basic research on antibiotics, whereas ampicillin and cefotaxime were chosen as experimental groups because of their widespread clinical use. The results showed that compared with the results of E001 (*bla*_TEM_), E002 (*bla*_CTX-M_), E003 (*bla*_KPC_), and E004 (*bla*_SHV_) without the action of antibiotics, the protein phosphorylation of E001 (*bla*_TEM_), E002 (*bla*_CTX-M_), and E004 (*bla*_SHV_) induced by 1/4 MIC penicillin G and cefotaxime sodium significantly increased (*p* < 0.0001). However, the phenomenon of protein phosphorylation promotion can be inhibited by 100 μg/mL of *I. tectorum* extract and closantel (132.5 μg/mL) (*p* < 0.0001). While with *E. coli* pretreated with 50 μg/mL of *I. tectorum* extract, only the protein phosphorylation induced by 1/4 MIC of cefotaxime sodium was inhibited (*p* < 0.0001). These results suggest that sublethal penicillin or cefotaxime can be used as signaling molecules to activate the TCSS, and this phenomenon can be inhibited by *I. tectorum* extract and closantel. In addition, under the action of 132.5 μg/mL of closantel and 100 μg/mL of *I. tectorum* extract, the mRNA levels promoted elimination (*p* < 0.0001).

Furthermore, we identified the two active components in the *I. tectorum* extract as irigenin and tectorigenin through TLC and LC-MS and determined their interaction sites with *E. coli* by molecular docking calculations. We found that these two substances have direct interactions with histidine residues 222 and 243, respectively (Figure 5). Therefore, we hypothesize that irigenin and tectorigenin may exhibit a competitive-inhibition relationship with phosphate groups. When irigenin and tectorigenin bind to histidine residue through Pi-Pi Stacked or Carbon Hydrogen Bond interactions, they cannot be activated by phosphate groups, thereby reducing the expression of ESBL genes (Figure 6).

At present, anti-β-lactamase drugs, such as sulbactam, tazobactam, and clavulanic acid, can bind to β-lactamase and make it inactive, but have no function in inhibiting the expression of the ESBL gene [38,39,40]. Closantel, which is a veterinary drug for the treatment of liver fluke disease in animals, can specifically inhibit the phosphorylation of HK and has great toxicity and side effects. In this study, we selected closantel as the positive control and *I. tectorum* extract as a candidate HK inhibitor. This study found that 100 μg/mL of *I. tectorum* extract significantly reduced the phosphorylation of HK and the levels of *bla*_TEM_, *bla*_CTX-M_, and *bla*_SHV_ mRNA, which were induced by 1/4 MIC of penicillin G and cefotaxime sodium. However, 50 μg/mL of *I. tectorum* extract significantly reduced HK phosphorylation and the levels of *bla*_TEM_, *bla*_CTX-M_, and *bla*_SHV_ mRNA, which were induced by 1/4 MIC cefotaxime sodium. The results showed that *I. tectorum* extract can inhibit β-lactamase expression at the gene level and enhance the antibiotic susceptibility of multidrug-resistant *E. coli*. Therefore, the *I. tectorum* extract has the potential to be used as a novel HK inhibitor.

## 4. Materials and Methods

### 4.1. Drug-Resistant E. coli

We used a total of 10 strains of β-lactam antibiotic-resistant *E. coli* in the entire experiment, all of which were donated by the Department of Microbiology, Zhejiang University School of Medicine, and preserved for research purposes. The quality control strains *E. coli* ATCC25922 and P. aeruginosa ATCC27853 were purchased from the China Institute of Food and Drug Identification (Hangzhou, China). A bacterial genomic DNA extraction kit (GK0122, Jierui, Shanghai, China) was used to extract the genomic DNA of drug-resistant *E. coli*, and the ESBLs genotypes (*bla*_TEM_, *bla*_CTX-M_, *bla*_KPC_, and *bla*_SHV_) were detected by polymerase chain reaction (PCR) [41]. The PCR (Applied Biosystems, Carlsbad, CA, USA) amplification conditions are as follows: heated to 94 °C for 5 min, repeated 30 times with a cycle of 94 °C for 30 s, 45 °C for 30 s, and 72 °C for 1 min, and finally kept at 72 °C for 10 min. The primers used for the PCR amplification are listed in Supplementary Materials Table S1. Drug-resistant strains containing only one ESBLs gene were screened in subsequent experiments.

### 4.2. Preparation of I. tectorum Extract

*I. tectorum* was collected from Huanglong Cave in May. Total flavonoids were extracted by a previously published macroporous resin method [42]. In brief, 200 g of dried rhizomes of *I. tectorum* were crushed and heated with seven times the volume of 80% ethanol three times for 1 h each time. After filtration, the filtrate was merged, the pressure was reduced to recover the ethanol, it was concentrated, and then it was dried for later use. The D101 macroporous resin (20070118, Kelong, Chengdu, China) was weighed and soaked in 95% ethanol for 24 h. After it had fully expanded, it was loaded into a chromatographic column and washed with distilled water until there was no alcohol odor [43,44]; 27 g of the ethanol extract was dissolved in 300 mL distilled water and subjected to resin column chromatography. The column bed was washed twice with 70% ethanol at a flow rate of 2 mL/min. The eluent was subjected to vacuum drying (DZG-6020, SENXIN, Shanghai, China) to obtain the crude extract of flavonoids, which was dried and stored for future use.

### 4.3. Determination of Antibacterial Activity of I. tectorum Extract

*I. tectorum* extract was diluted in multiple ratios to a series of appropriate concentrations, and 1 mL of each solution was added to the test tubes. Each extract at different concentrations was repeated three times, and a 3 M-H liquid medium without the extract was used as a blank control. One milliliter of bacterial suspension (*bla*_SHV_) was added to each test tube, adjusted to a 0.5 McFarland concentration (WGZ-20B, INESA, Shanghai, China), and diluted with 10 times the volume. After culturing on a shaking table (ZWY-240, ZHICHENG, Shanghai, China) at 37 °C for 18–24 h, the bacteria were inoculated onto an ordinary plate and cultured in a 37 °C incubator (SHP-250, SENXIN, Shanghai, China) for 18–24 h to observe their growth. The minimum inhibitory concentration (MIC) of bacteria with no growth was considered the minimal inhibitory concentration. The experimental results are listed in Supplementary Materials Table S2.

### 4.4. Determine the Effect of I. tectorum Extract on the Antibacterial Activity of Ampicillin

M-H agar plates containing a series of concentrations of ampicillin (130401, HaoTian, Hangzhou, China) and a series of concentrations of *I. tectorum* extract (96.25 μg/mL, 19.25 μg/mL, and 3.850 μg/mL) were prepared. A fresh culture of the drug-resistant bacteria (*bla*_SHV_) was prepared in a 0.5 McFarland concentration bacterial suspension and then diluted at 1:10 to make a final concentration of 10^7^ CFU/mL. Continuous streaky inoculation was performed on the prepared M-H agar plate and cultured in a biochemical incubator at 37 °C for 18–24 h to observe whether the bacteria grew. The MIC of ampicillin sodium against drug-resistant *E. coli* was determined after treatment with *I. tectorum* extract and ampicillin sodium.

### 4.5. Determine the Effect of I. tectorum Extract on the Reversal Rate of Drug Resistance in E. coli

Ten strains of *E. coli*, E001 (*bla*_TEM_), E002 (*bla*_CTX-M_), E003 (*bla*_KPC_), E004 (*bla*_SHV_), E005 (*bla*_TEM_), E006 (*bla*_CTX-M_), E007 (*bla*_CTX-M_), E008 (*bla*_TEM_), E009 (*bla*_SHV_), and E010 (*bla*_TEM_), were selected and inoculated on LB solid medium and incubated at 37 °C for 18–24 h. A single colony was incubated in l0 mL LB liquid medium at 37 °C and oscillated at 220 rpm overnight. The bacterial solution (200 µL) was absorbed and inoculated into a fresh 20 mL of LB liquid medium and incubated at 37 °C and 220 rpm until the bacteria grew to a logarithmic phase. And the optical density is 0.6–0.8 (1 × 10^8^) at 600 nm (OD600) (NANODROP ONE, Waltham, MA, USA). Then, *I. tectorum* extract (working concentrations were 100.0 μg/mL, 50.0 μg/mL, and 25.0 μg/mL) was added and co-incubated for 0.5 h. Microtube dilution methods were applied to test the changes in the MIC of *E. coli*. The sensitivity or resistance of the strain to penicillin G (20121011, Zeheng, Hangzhou, China) and cefotaxime sodium (201306, Zeheng, Hangzhou, China) was determined according to CLSI 2017. The rate of reversal of antibiotic resistance was then calculated. *E. coli* ATCC 25922 and *P. aeruginosa* ATCC 27853 were used as the quality control organisms.

### 4.6. Determine the Effect of I. tectorum Extract on the Transcription of ESBLs Genes

Real-time quantitative polymerase chain reaction (RT-qPCR) (AK2001, TaKara, Dalian, China) was performed to determine the effects of 1/4 MIC penicillin G and cefotaxime sodium on *E. coli* E001, E002, E003, and E004 gene expression and the inhibitory effect of *I. tectorum* extract. The transcription levels of ESBLs genes in *E. coli* were detected by RT-qPCR (7500 real time PCR system, Singapore) after the strains were co-incubated with *I. tectorum* for 0.5 h. The reaction conditions for RT-qPCR were as follows: heating to 95 °C for 5 min, repeated 40 times with a cycle of 94 °C for 5 min, repeated 30 times with a cycle of 94 °C for 30 s, 45 °C for 30 s, and 72 °C for 1 min, and, finally, maintained at 72 °C for 10 min.

### 4.7. Determine the Effect of I. tectorum Extract on the Histidine Kinase Phosphorylation

*E. coli* E001, E002, E003, and E004, which contain only one ESBL gene, were co-incubated with *I. tectorum* extract for 0.5 h. Then, 1/4 MIC penicillin G or cefotaxime sodium was added and incubated at 37 °C for 1 h, followed by centrifugation at 5000 rpm for 15 min. The resulting bacterial precipitate was rinsed with normal saline to remove interference from the extract and antibiotics. The lysate was added to the bacterial precipitate and incubated in an ice bath for 30 min, according to the instructions of the Bacterial Protein Phosphorylation Enrichment Kit (BestBio, Nanjing, China). The cells were then disrupted by ultrasonication (300 w; 3 s each at 3 s intervals; 15 min) (SCIENTZ-IID, Ningbo, China) and centrifuged at 12,000 rpm at 4 °C for 5 min to collect the supernatant. The protein concentration in the supernatant was determined using a BCA Kit (Generay, Shanghai, China). The protein samples were adjusted to 0.5 mg/mL with the bacterial lysate, and the phosphorylated protein was enriched using immobilized metal ion affinity chromatography (Bestbio, Nanjing, China). Bacterial protein phosphorylation levels were detected by a Protein Phosphorylation Detection Kit (Sangon, Shanghai, China) and a spectrometer (Bio-Rad, Hercules, CA, USA). The concentration of phosphorylated protein in each sample was determined according to the standard curve of protein phosphorylation at 600 nm (OD600). Compared to the results for E001, E002, E003, and E004 without the action of the *I. tectorum* extract, we analyzed the effect of the *I. tectorum* extract on the levels of histidine kinase phosphorylation. The HK inhibitor closantel (132.5 μg/mL) was used as the positive control.

### 4.8. Analysis of the Components in I. tectorum Extract

Compared with standard flavonol (SLCC9071, SIGMA, Shanghai, China), the absorbance was measured at a wavelength of 266.5 nm after a series of concentrations were configured and the standard curve was drawn. The total flavonoid content of the *I. tectorum* extract was calculated according to the standard curve. The *I. tectorum* extract, flavonol standard, and crude drug extraction solutions were tested by TLC, and the sites of the unfolding bands were recorded and compared. Finally, the *I. tectorum* extract was analyzed by LC-MS (Zeno TOFTM 7600, SCIEX, Washington D.C, WA, USA) to determine the components of the flavonoids.

### 4.9. Molecular Docking

We retrieved and downloaded the 3D structure of histidine kinase from *E. coli* in PDB format from the UniProt public protein database “https://www.uniprot.org/ (accessed on 5 December 2024)”. And we downloaded the 3D structures of irigenin and tectorigenin in SDF or MOL format from PubChem “https://pubchem.ncbi.nlm.nih.gov/ (accessed on 5 December 2024)” and the Traditional Chinese Medicine Systems Pharmacology Database and Analysis Platform (TCMSP, “https://www.tcmsp-e.com/#/database (accessed on 5 December 2024)”), respectively. Then, we used Discovery Studio 2019 software to modify the protein receptor and small molecule ligands, set up the docking box, and perform molecular docking.

### 4.10. Statistical Methods

The transcript levels of ESBL genes and histidine kinase phosphorylation were statistically analyzed using the GraphPad Prism 8 statistical software *t*-test, and *p* < 0.05 indicates statistical significance.

## 5. Conclusions

The present study shows that *I. tectorum* extract may serve as a HK inhibitor to alleviate bacterial resistance. We found that 100 μg/mL or 50 μg/mL of *I. tectorum* extract significantly reversed the resistance of *E. coli* strains to β-lactam antibiotics such as penicillin G and cefotaxime sodium and reduced ESBL gene expression and histidine kinase phosphorylation levels in the TCSS induced by 1/4 MIC of antibiotics. This study of the effects of flavonoid substances in Chinese herbal medicine on bacterial metabolism can provide support for further research on the properties, efficacy, and antibacterial activity of flavonoid active ingredients and provide a theoretical basis for the synthesis of new antibacterial drugs and drug screening.

## Figures and Tables

**Table 1:** The MIC of different concentrations of extract and ampicillin simultaneously.

	Concentration (μg/mL)	Ampicillin MIC (μg/mL)	R/S
*I. tectorum* extract	96.3	<15.6	S-I
	48.1	125.0	R
	19.3	2000.0	R
	3.9	2000.0	R
	0.0	2000.0	R

Drug sensitivity results were interpreted according to CLSI2017; MIC ≤ 8 was determined to be S, and MIC ≥ 32 was determined to be R. S: susceptible. I: intermediate. R: resistance.

## Data Availability

Data will be made available on request.

## Supplementary Materials

The following supporting information can be downloaded at https://www.mdpi.com/article/10.3390/molecules30030663/s1: Figure S1: Standard curve for determination of flavonoid content; Table S1: The primers of *bla*_TEM_, *bla*_CTX-M_, *bla*_KPC_, and *bla*_SHV_ genes; Table S2: The MIC values of the *I. tectorum* extract to drug-resistant *E. coli*.

## Abbreviations

ARGs	Antibiotic-resistance genes
CARSS	China Antimicrobial Resistance Surveillance System
CLSI	Clinical and Laboratory Standards Institute
*E. coli*	*Escherichia coli*
ESBLs	Extended-spectrum beta-lactamases
HK	Histidine kinase
*I. tectorum*	*Iris tectorum*
LC-MS	Liquid chromatograph mass spectrometer
MIC	Minimum inhibitory concentration
*P. aeruginosa*	*Pseudomonas aeruginosa*
qPCR	Quantitative polymerase chain reaction
RRP	Response regulator protein
RT-qPCR	Reverse transcription quantitative polymerase chain reaction
SDGs	Sustainable Development Goals
SK	Serinepthreonine kinase
TCSS	Two-component signaling system
TK	Tyrosine kinase
TLC	Thin-layer chromatography
