# Supplementary material for: Small Molecules from Medicinal Plant Iris tectorum as Histidine Kinase Inhibitor to Resensitize β-Lactam-Resistant Escherichia coli
Source: Molecules. 2025 Feb 3;30(3):663. doi: 10.3390/molecules30030663 (PMC11820157; doi:10.3390/molecules30030663)
Supplement: Supplementary file 1 [file molecules-30-00663-s001.zip › molecules-3430006-supplementary.pdf]

Supplementary Table S1 The primers of *bla*$_{TEM}$, *bla*$_{CTX-M}$, *bla*$_{KPC}$, *bla*$_{SHV}$ genes

Primers	Sequence（5′-3′）	Product size（bp）
bla$_{TEM}$	F：TCGGGCAAATGTGCG	972
	R：TGCTTAATCAGTGAGGCACC	
bla$_{CTX-M}$	F：GTTACAGCCCTTCGGCGATGATTC	881
	R: GCGCATGGTGACAAAGAGAGTGCA	
bla$_{SHV}$	F：GCCGGGTTATTTTATTTGTCGC	1017
	R：TCTTTCCGATGCCGCCGCCAGTCA	
bla$_{KPC}$	F：GCTACACCTAGCTCCACCTCC	1050
	R：TCAGTGCTCTACAGAAAACC	
16S rRNA	F：AGAGCAAGCGGACCTCATAAA	113
	R：AACGTATTCACCGTGACATTCTG	

F: Forward primer; R: Reverse primer

Supplementary Table S2 The MIC values of *I. tectorum* extract to drug-resistant *E. coli*

	Sample concentration (μg/mL)							MIC
	385.0	192.5	96.2	48.1	24.1	12.0	negative control	
Drug-resistant *E. coli*	-	-	+ +	+ + +	+ + +	+ + +	+ + +	192.5

Note: The above data are all the average values of three repeated experiments; '+' indicates a very small amount of bacterial growth; '++' indicates a small amount of bacterial growth; '+++' indicates the growth of a large number of bacteria; '-' indicates sterile growth

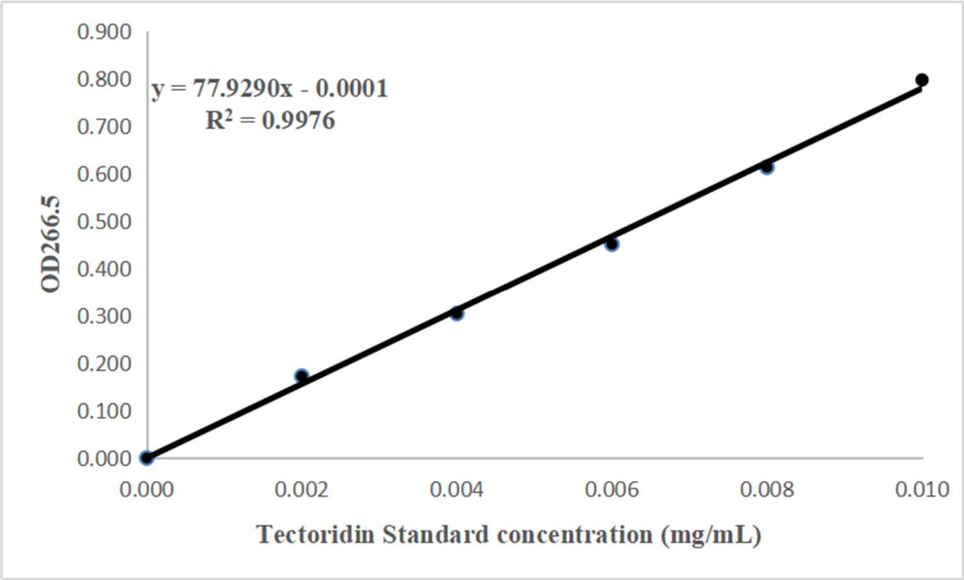

Supplementary Figure S1 Standard curve for determination of flavonoid content.
